# Uremic Leontiasis Ossea in a Patient With End-Stage Renal Disease in Hemodialysis

**DOI:** 10.7759/cureus.11060

**Published:** 2020-10-20

**Authors:** Karim Nasra, Mario Dervishi, Shuo Li, Denis R Lincoln

**Affiliations:** 1 Radiology, Ascension Providence Hospital, Southfield, USA; 2 Radiology, American University of the Caribbean School of Medicine, Cupecoy, SXM

**Keywords:** uremic leontiasis ossea, esrd, secondary hyperparathyroidism, radiology

## Abstract

Uremic leontiasis ossea is a rare condition, reported in patients with severe renal disease. Patients present with progressive enlargement of facial bones - in particular, the maxillary and mandibular bones. Rarity of this condition leaves clinicians puzzled on initial evaluation and management. Herein, we present a 31-year-old man diagnosed with uremic leontiasis ossea. The report aims to review the pathophysiology of the condition as described in the literature, the patient presentation and imaging modalities used to investigate, and classical findings seen in patients with uremic leontiasis ossea. Finally, we briefly touch base on the reported regimens used to prevent and manage this condition.

## Introduction

Leontiasis ossea is a term used to describe a number of conditions that affect a patient's facial and cranial bone structures, and it is colloquially referred to as a lion's face [1]. Uremic leontiasis ossea (ULO) is a severe, and rare form of leontiasis ossea, with characteristic overgrowth of cranial bones secondary to uncontrolled hyperparathyroidism in end-stage renal disease (ESRD) patients [1, 2].

The physical appearance of unilateral or bilateral overgrowth of the maxillary bone is most commonly prominent in ULO patients. However, initial presentation of the facial deformities may not be as obvious and not always clearly distinguishable. Facial swelling and bilateral or unilateral jaw pain may be part of the initial complaints. Progressive expansion and bone overgrowth of the facial cranial structures can gradually lead to multiple complications, including exophthalmos and complete visual loss due to compression of the optic nerve [3]. Other nearby anatomical structures in the head and neck area can also be affected by overgrowth mass effect and present symptomatically.

Though only a relative few cases have been reported, the severity of the disease can cause significant distress to patients. No current or standard therapies exist in management of ULO and the best measurement is strict dialysis and medical control of secondary hyperparathyroidism. In this report we present a case of a 31-year-old patient who was diagnosed with uremic leontiasis ossea.

## Case presentation

A 31-year-old man with a past medical history of ESRD of unknown etiology, on hemodialysis (HD) since 2004, presented to the emergency department (ED) complaining of missed dialysis. He also complained of shortness of breath with exertion, orthopnea, as well as mild chest pain. Fluid overload was suspected due to the patient's missed dialysis. Further laboratory evaluation showed severe hyperkalemia (6.2 mmol/L), high blood urea nitrogen (104 mg/dL), creatinine (17.2 mg/dL), phosphorus (7.1 mg/dL) and normal calcium (9 mg/dL). HD treatment was initiated and further post-HD evaluation showed improvement of the patient's overall status.

During his admission, the patient complained of right-sided facial swelling which had been gradually worsening for the past three weeks, with increased severity in the last three days. He complained of difficulty chewing. Right-sided upper and lower dental pain was also noted. He denied any headaches, fever, cough, nausea, vomiting or naso-oropharyngeal discharge.

As per patient's report, he had a similar presentation affecting his left jaw in 2012. At that time, the patient recalls undergoing a left jaw “surgical procedure” at an out-of-state, outside institution but has no recollection of imaging evaluation or indication for the procedure.

**Figure 1:**
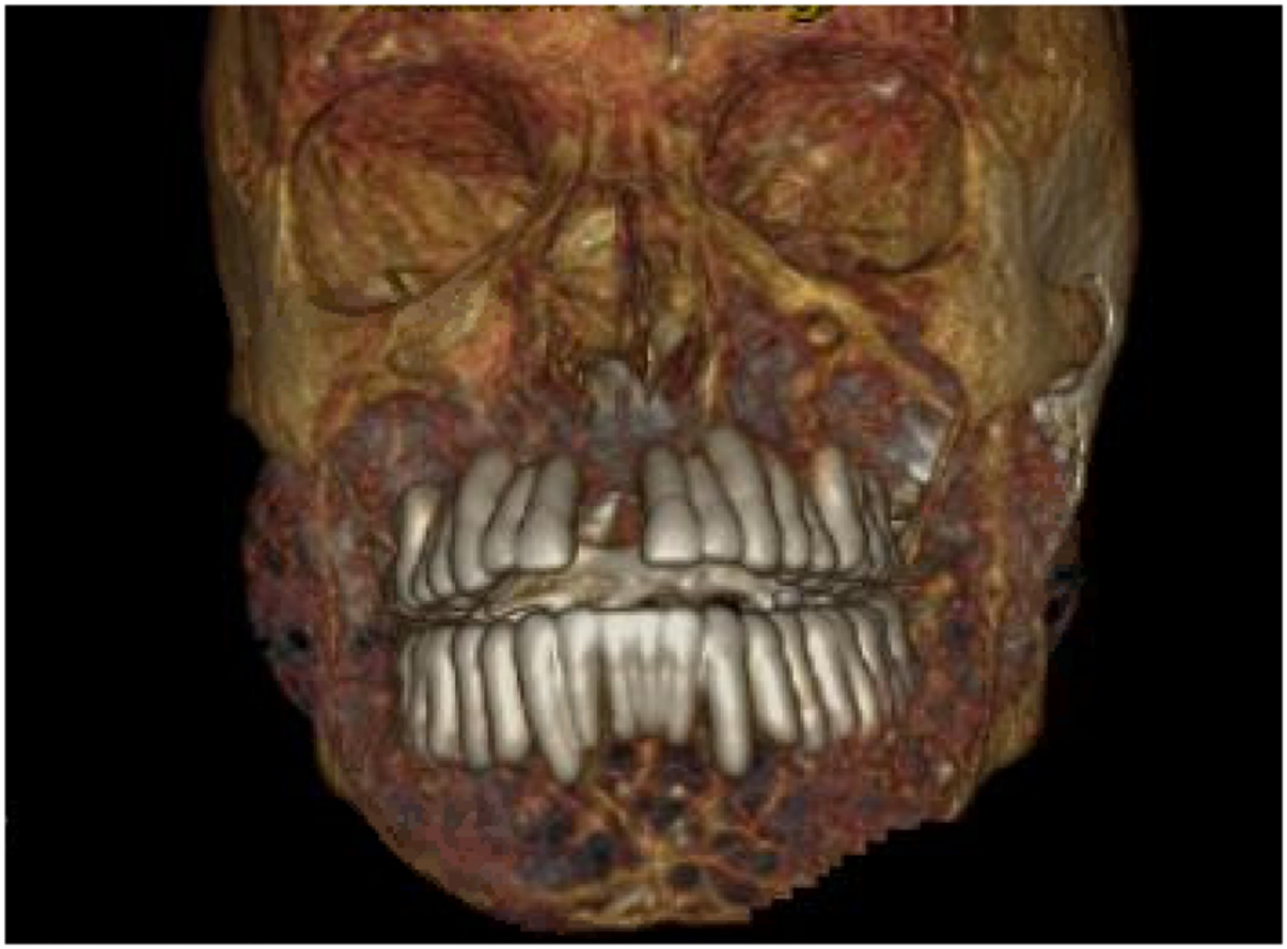
3D rendering of the computed tomography showing abnormal appearance of the maxilla, mandible and the rest of facial bones.

**Figure 2:**
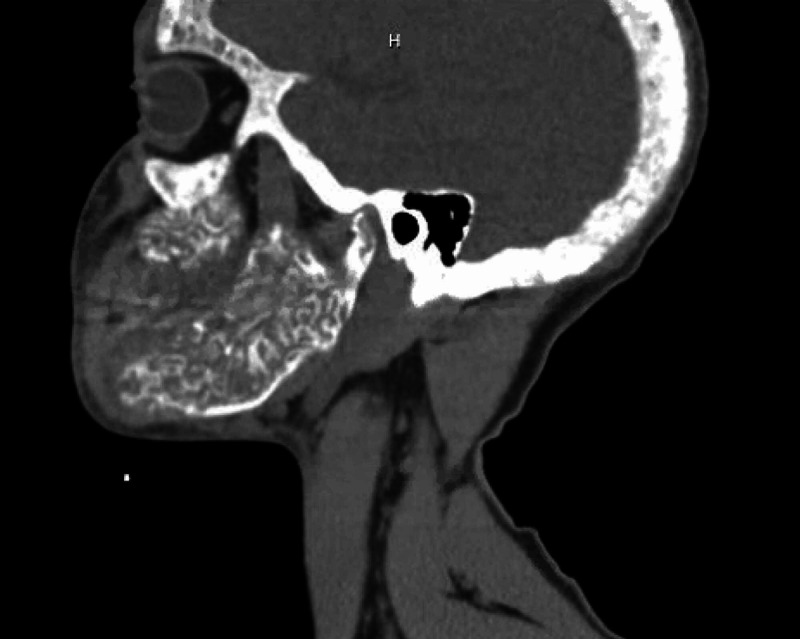
Sagittal view - Non-contrast CT showing markedly expanded mandible with prominent dense trabeculae. Calvarium appears abnormal, thickened with a smoking appearance.

**Figure 3:**
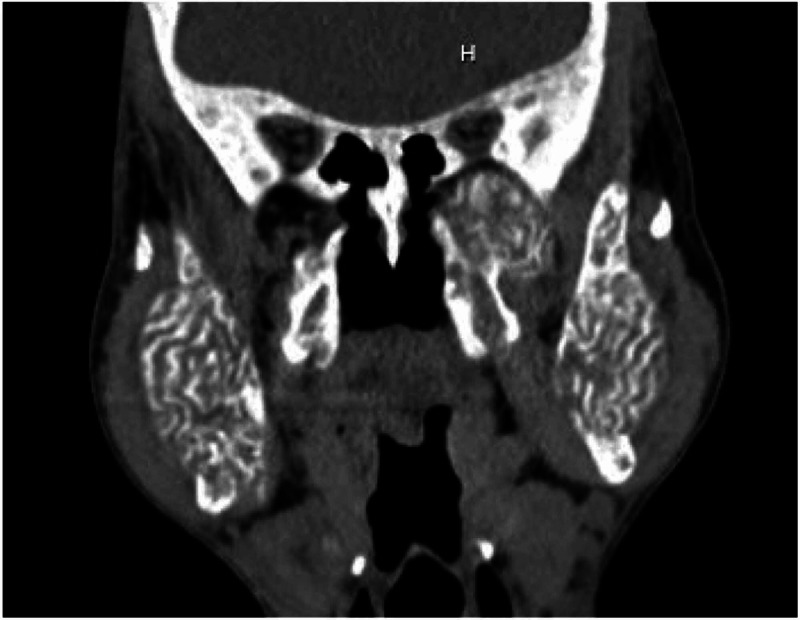
Coronal view - Non-contrast CT showing expanded mandible with prominent dense trabeculae.

**Figure 4:**
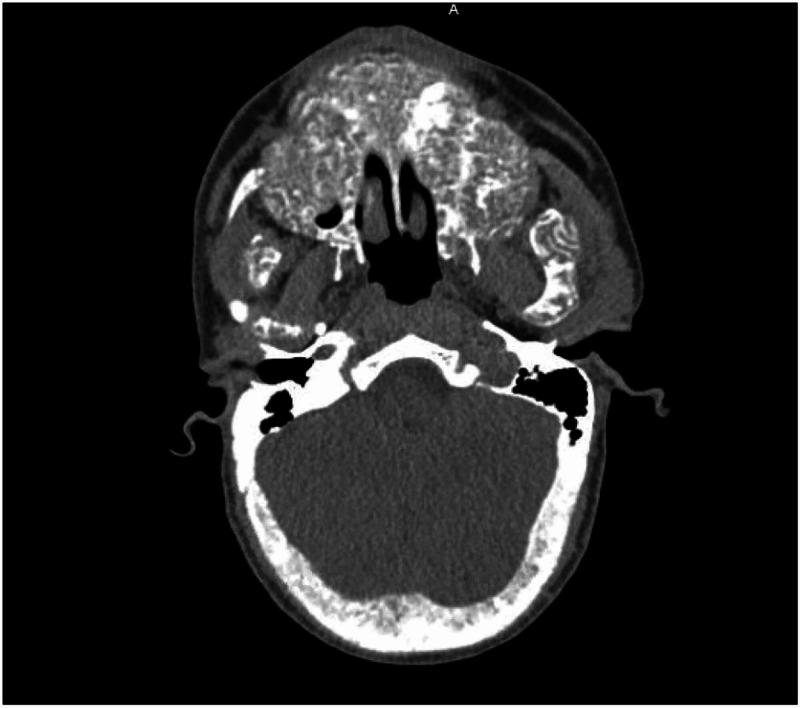
Axial view - Non-contrast CT showing expanded mandible with prominent dense trabeculae. Involvement of alveolar margin is also noted, with worse findings on the left side.

A computed tomography (CT) of soft tissue of the neck and mandible without contrast was ordered for further evaluation of the facial swelling and associated symptoms. Imaging findings showed abnormal appearance of the skull, mandible, and facial bones. A markedly expanded mandible with prominent dense trabeculae was noted. Similar appearance involving the alveolar margin was also noted, with worse findings on the left side. The calvarium appeared to be abnormal, thickened with a smoking appearance throughout the medullary cavity (Figures 1-4).

**Figure 5:**
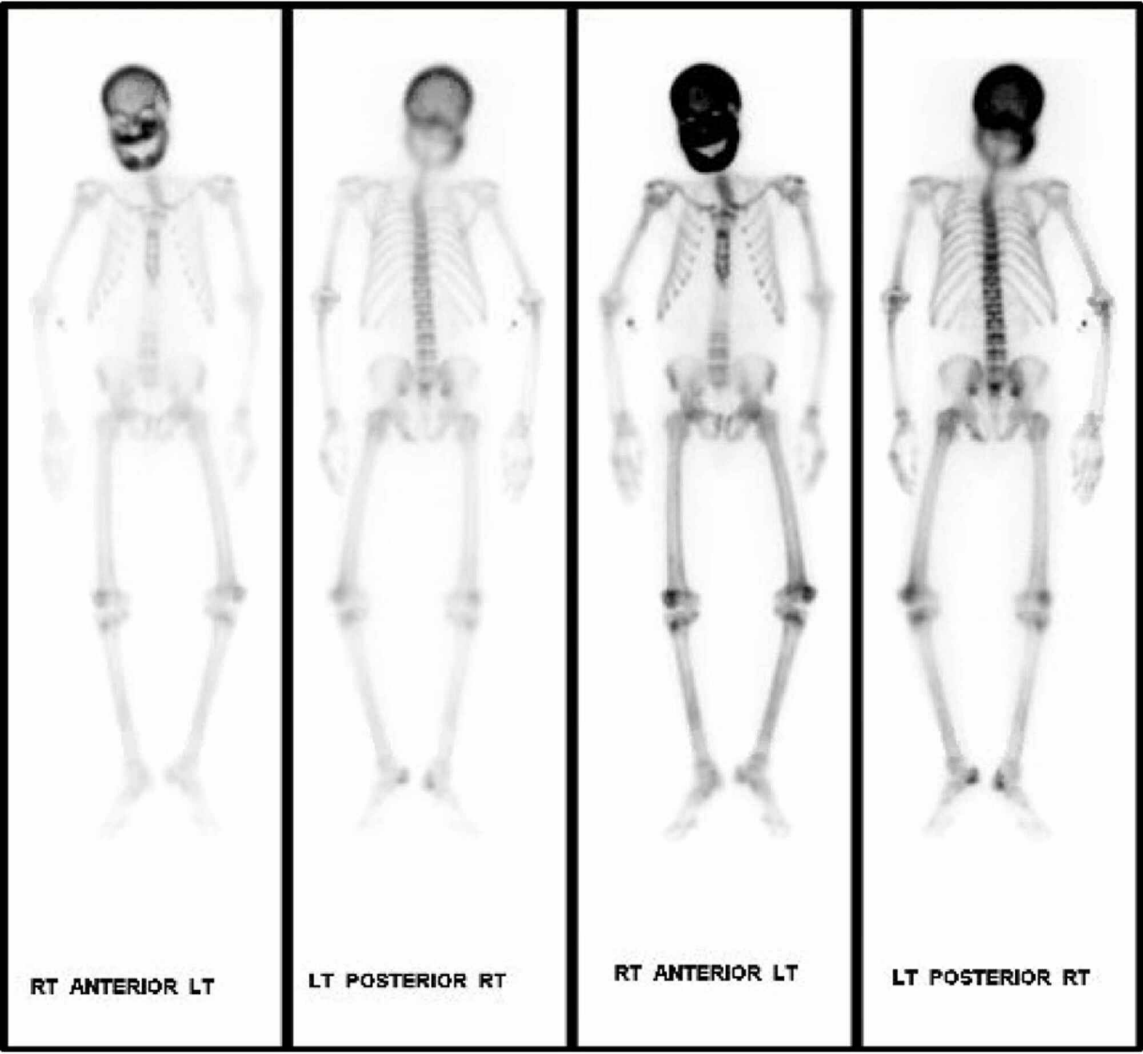
Technetium 99m methylene diphosphonate (MDP) bone scan showing increased activity throughout the calvarium, facial bones, primarily in the alveolar margin, and throughout the mandible.

A nuclear medicine (NM) bone scan (Technetium 99m methylene diphosphonate (MDP)) was ordered for further evaluation of the CT findings. The bone scan showed increased activity throughout the calvarium, facial bones, primarily in the alveolar margin, and throughout the mandible (Figure 5).

After a literature search, the imaging findings fit the diagnosis of uremic leontiasis ossea, a form of severe bone remodeling that prevails in patients with chronic kidney disease, secondary hyperparathyroidism, and renal osteodystrophy.

After the initial dialysis treatment, the patient refused to stay for further management of his ESRD and the plan of care of uremic leontiasis ossea and left against medical advice. Further evaluation of this patient including parathyroid hormone (PTH) levels were unable to be obtained at this time.

## Discussion

Uremic leontiasis ossea is a chronic, infrequently reported condition resulting from secondary hyperparathyroidism in ESRD patients [2, 3]. Poorly managed chronic renal insufficiency leads to decreased calcium resorption and phosphate excretion, activating the parathyroid gland. In addition, decreased activation of Vitamin D in the kidney leads to increased PTH secretion. Excess PTH leads to unbalanced increased activity of osteoclasts and osteoblasts which results in increased unmineralized bone contributing to the ULO; the same pathogenesis is seen in renal osteodystrophy [2, 4, 5].

ESRD patient’s non-adherent to the hemodialysis regimen and medical treatment are likely most susceptible to uremic leontiasis ossea. Such non-compliance to hemodialysis is also seen in our ESRD patient, leading up to his ULO presentation due to possible chronic metabolic derangements and secondary hyperparathyroidism [3-5].

Patients present with enlarged mandible and maxilla and other cranial bone distortions [6, 7]. Besides the obvious disfigurement of the patient, structural changes of the craniofacial bones can lead to multiple complications. Compression of local structures including nerves and vasculature, and complete visual loss due to compression of the optic nerve are some of the most serious complications [3]. Enlargement of the facial structures is not always obvious and as seen in our patient, initial complaints can be jaw pain and or facial swelling. A facial photograph of the patient was not obtained as he left against medical leave and there were no prior photographs to assess for obvious facial deformations and changes throughout the years.

CT scan and NM bone scans have been the modalities of choice to investigate ULO. Select, older publications also document use of head X-ray and MRI for research purposes [8]. For clinical purposes, CT and NM bone scans were deemed sufficient to assess the extent of our patients' condition. Classic findings, on CT imaging, seen in patients with ULO are hypertrophy and hyperostosis of maxillary bone, possible obliteration of the maxillary sinuses as well as diffuse enlargement of the mandible [6-8]. CT findings found in our patient fit that description as well.

NM bone scintigraphy use in ULO evaluation has been reported in a few cases and findings show increased bone reuptake demonstrating various skeletal characteristics of renal osteodystrophy [6-9]. Our bone scintigraphy showed the same findings as well.

The use of facial bone biopsies has been documented to further investigate the condition. Reported biopsy findings in ULO have shown extensive bone marrow fibrosis with increased areas of woven bone and mixture of osteoclasts and osteoblasts, consistent with osteitis fibrosa [8]. It is very possible that the “surgical procedure” our patient reports in 2012 at the outside institution could have been a bone biopsy. As demonstrated in our case, the patient’s clinical presentation congruent with image findings seen on CT and NM bone scan is sufficient to make the diagnosis of ULO and bone biopsy may not be necessary.

To date, there is no current standardized approach to diagnosis and treatment of ULO due to the rarity of the condition. Management revolves around strict control of ESRD patients with hemodialysis and medical therapy to prevent metabolic derangements and optimize parathyroid hormone (PTH) secretion [3]. Hence, compliance to hemodialysis and the medical regimen is crucial for prevention of development of ULO. In patients already diagnosed with ULO, medical therapy to control PTH and hemodialysis remain the main treatment. In a few instances, parathyroid resection with auto transplantation has been documented as an effective therapy as it appears that this halts bone remodeling [10]. No cases of surgical resection of bone exist though a multidisciplinary approach and intervention should be considered if life-threatening complications arise due to the condition.

## Conclusions

In conclusion, ULO is a rare condition that may be prudent to include in the differential diagnosis in patients with ESRD, complaining of jaw pain and progressive facial and bone deformities. History of extensive renal disease with chronic metabolic derangements and high PTH as well as classic findings in CT of the head and neck and bone scintigraphy are sufficient to make the diagnoses. Biopsy of the maxillary bone can be a last resort option to aid in diagnosis but most likely is unnecessary. Strict medical management of metabolic derangements, prevention of secondary hyperparathyroidism and adherence to hemodialysis are the best possible options to prevent this condition.
